# Serous atrophy of bone marrow manifested as subcutaneous and visceral fat depletion on CT: two case reports

**DOI:** 10.3389/fonc.2026.1750230

**Published:** 2026-03-10

**Authors:** Zheng Han, Jiang-Feng Pan, Liang Hu

**Affiliations:** Department of Radiology, Jinhua Municipal Central Hospital, Jinhua, Zhejiang, China

**Keywords:** computed tomography, fat tissue, magnetic resonance imaging, nutritional status, serous atrophy of bone marrow

## Abstract

Serous atrophy of the bone marrow (SABM) is primarily caused by the breakdown of bone marrow fat due to the depletion of body fat reserves. SABM is linked to poor nutritional status or cachexia in advanced cancer stages and exhibits distinct characteristics on magnetic resonance imaging (MRI). However, these adverse conditions create challenges for undergoing MRI, thereby delaying timely diagnosis. This case report details changes in body fat tissue observed through CT scans during the progression of two SABM cases. Two patients were elderly males, and both had undergone chemotherapy and immunotherapy following gastric cancer resection. The MRI scans of both patients revealed the characteristic “flip-flop” effect, in which T1WI appears to show fat suppression, while fat-suppressed T2WI appears to show fat without suppression. This finding differs from other bone marrow disorders and led to a diagnosis of SABM. Previous longitudinal CT analyses indicated that both subcutaneous and visceral fat tissues were not only reduced but had ultimately disappeared, resulting in a fluid-like density in the CT images of two cases. This finding indicates the depletion of body fat reserves. Furthermore, it suggests a significant deterioration in nutritional status and raises strong suspicion regarding the potential development of SABM.

## 1 Introduction

Serous atrophy of bone marrow (SABM), also referred to as starvation of bone marrow, is a lesion characterized by the atrophy of bone marrow fat and the accumulation of extracellular mucopolysaccharides, including hyaluronic acid (1, 2). SABM is primarily linked to anorexia nervosa (3). The pathogenesis of SABM is not fully understood; however, existing evidence suggests a close association with prolonged malnutrition states, such as anorexia nervosa and cachexia (4, 5). Advancements in cancer treatment have markedly extended patient survival times. Patients with advanced-stage cancer often experience a prolonged state of increased energy expenditure, which is exacerbated by insufficient nutritional intake. This combination can result in malnutrition, making them particularly susceptible to SABM. SABM is primarily diagnosed through the characteristic “flip-flop” findings observed in magnetic resonance imaging (MRI) (4, 6, 7), which are critical for accurate identification of the condition. However, patients who are severely malnourished rarely undergo MRI scans due to their poor health, which complicates the diagnosis of SABM in these populations. Early identification facilitates nutritional interventions and fracture prevention (5). Computed tomography (CT) scans are commonly used for postoperative follow-up in cancer patients; moreover, they provide valuable information regarding body composition. The body composition measured by CT scans, including the visceral and subcutaneous fat indices, as well as the skeletal muscle index, is closely associated with the body’s nutritional status (8–10). Therefore, these follow-up changes in the indices may be relevant with the development of SABM, suggesting a possible avenue for early diagnosis.

This case report investigates the characteristics of changes in body composition based on CT scans of two patients diagnosed with SABM.

## 2 Case report

Case 1:A 68-year-old male was admitted to the hospital three years after undergoing surgery for gastric cancer. The patient underwent a total gastrectomy for gastric malignancy, with a pathology report indicating high-grade adenocarcinoma at stage PT3N3bM0 in April 2021. Seven cycles of chemotherapy utilizing the TX regimen (paclitaxel 400 mg on day 1, capecitabine 1.5 g, bid, day 1 to day 14) were administered following surgery. A CT scan conducted in August 2023 suggested the presence of implantation metastasis in the pelvic cavity. In September 2023, tegafur was administered at a dosage of 20 mg twice daily, alongside sintilimab at a dosage of 100 mg for immunotherapy. At this time, the nutritional assessment indicated moderate malnutrition, characterized by a Body Mass Index (BMI) of 17.15 kg/m² and a Patient-Generated Subjective Global Assessment (PG-SGA) score of 7 points. He was readmitted in March 2024 for a nutritional assessment due to severe malnutrition, characterized by a BMI of 16.3 kg/m² and a PG-SGA score of 9 points.

**Figure 1:**
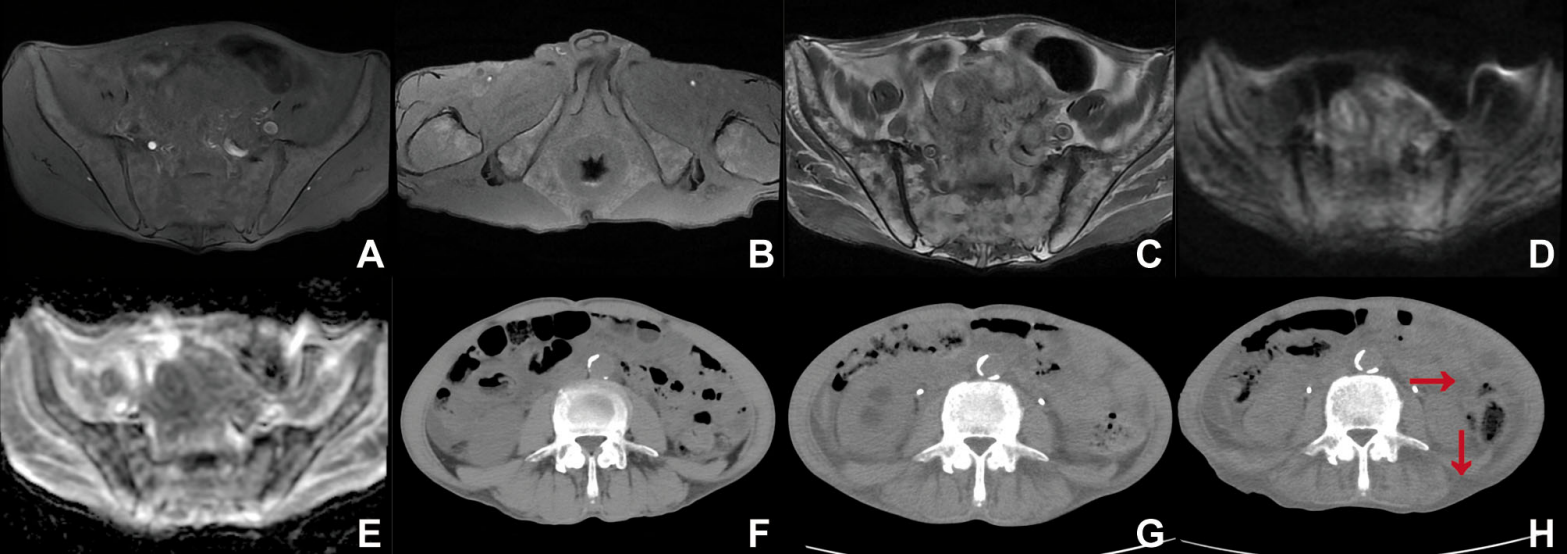
**(A)** T1WI (sacroiliac joint level), **(B)** T1WI (ischiorectal fossa level), **(C)** Fat-suppressed T2WI (T2WI-SPAIR), **(D)** DWI (b=800 s/mm^2^), **(E)** ADC map, **(F–H)** Abdominal computed tomography (CT) scans were conducted seven months ago **(F)**, three months ago **(G)**, and at present **(H)**, utilizing a window width (WW) of 350 and a window level (WL) of 40.

The MRI of the pelvis (March 2024): the T1-weighted imaging (T1WI) demonstrated the absence of high signal intensity in the abdominal cavity and subcutaneous fat region (**Figure 1A**), along with a small residual high signal in the ischiorectal fossa (**Figure 1B**). Fat-suppressed T2-weighted imaging (T2WI) exhibited high signal intensity in the abdominal cavity and the subcutaneous fat region (**Figure 1C**). The high signal intensity of fat on T1WI appeared suppressed (**Figures 1A, B**), while the fat signal on fat-suppressed T2WI did not exhibit suppression (**Figure 1C**). The pelvic bone marrow exhibited inhomogeneous iso-slightly low signal intensity on T1WI (**Figures 1A, B**) and inhomogeneous slightly high signal intensity on fat-suppressed T2WI (**Figure 1C**). Diffusion-weighted imaging (DWI) demonstrated iso-signal of the pelvic bone marrow (**Figure 1D**), while the apparent diffusion coefficient (ADC) map displayed a slightly heterogeneous high signal in the pelvic bone marrow (**Figure 1E**).

The abdominal CT scan evaluates the L3 skeletal muscle index, visceral fat index, and subcutaneous fat index. These indices are calculated by measuring the total cross-sectional area of skeletal muscle, visceral fat, or subcutaneous fat tissue (in cm²) on a single CT scan image at the level of the third lumbar vertebra. This area is then divided by the square of the patient’s height (in m²) to yield the final index values expressed in cm²/m². According to measurements obtained using SliceOmatic 5.0, the skeletal muscle index values were 41.22 cm²/m² seven months ago (August 2023) (**Figure 1F**), 40.02 cm²/m² three months ago (December 2023) (**Figure 1G**), and 37.88 cm²/m² at present (March 2024) (**Figure 1H**). Concurrently, the subcutaneous fat index was recorded as 0.62 cm²/m² (**Figure 1F**), 0 cm²/m² (**Figure 1G**), and 0 cm²/m² (**Figure 1H**), respectively, while the visceral fat index was 2.03 cm²/m² (**Figure 1F**), 0.34 cm²/m² (**Figure 1G**), and 0 cm²/m² (**Figure 1H**), respectively. The abdominal visceral and subcutaneous fat regions demonstrated a reduction (**Figures 1F–H**), accompanied by an increase in their densities, ultimately exhibiting a fluid-like density as indicated by the red arrow in the CT image (**Figure 1H**). There was a gradual atrophy of the lower back muscles accompanied by fatty infiltration (**Figures 1F–H**).

Case 2: A 68-year-old male patient was admitted to the hospital more than one year after undergoing surgical intervention for gastric cardia cancer. The patient was diagnosed one year ago (May 2022) with a carcinoma of the esophagus and cardia. The tumor was surgically excised, and pathologically confirmed as a poorly differentiated adenocarcinoma, classified as stage pT4aN3aM0. The patient received postoperative treatment with the SOX regimen (oxaliplatin (180 mg) and tegafur (60 mg) administered twice daily from days 1 to 14), and treatment was later adjusted to single-agent tegafur chemotherapy. In October 2023, retroperitoneal lymph node metastases were identified and subsequently treated with tislelizumab immunotherapy. The admission assessment (January 2024) indicated moderate malnutrition, characterized by a BMI of 19.5 kg/m² and a PG-SGA score of 6 points. He was readmitted to the hospital in May 2024, where the admission assessment revealed severe malnutrition, characterized by a BMI of 17.6 kg/m² and a PG-SGA score of 9 points.

**Figure 2:**
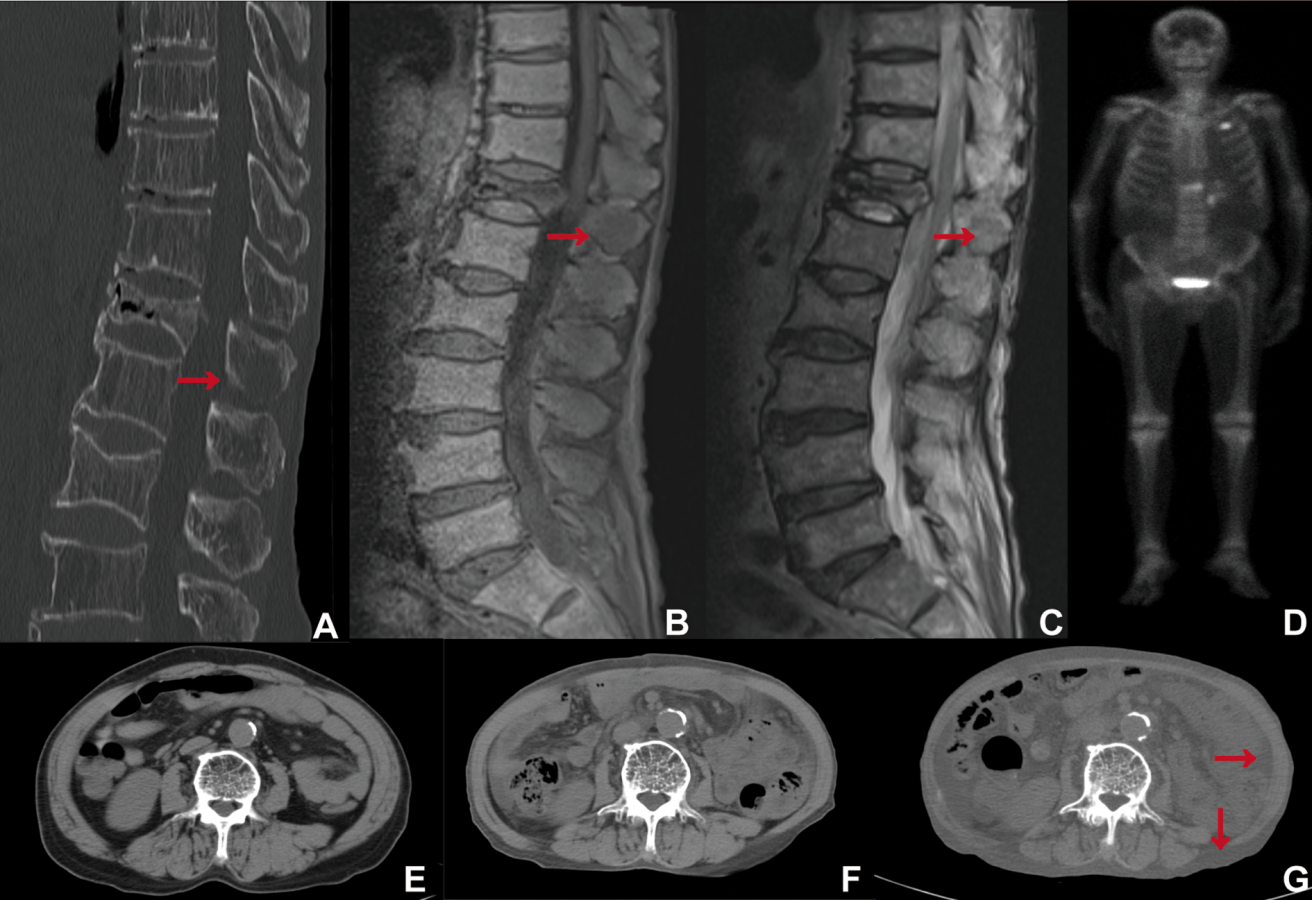
**(A)** Thoracolumbar spine CT (WW/WL 2600/800), **(B)** T1WI, **(C)** fat suppressed T2WI (T2WI-STIR), **(D)** Radionuclide bone scintigraphy, **(E–G)** Abdominal CT scans were performed 24 months prior **(E)**, 18 months prior **(F)**, and one month prior **(G)**, with a WW of 350 and a WL of 40.

A CT scan conducted in May 2024 revealed osteoporosis of the thoracolumbar spine, a compression fracture of the thoracic vertebral body at T12, and osteolytic bone metastases in the spinous process of T12, as indicated by the red arrow (**Figure 2A**). MRI revealed a loss of high signal intensity in the abdominal and dorsal subcutaneous fat region in T1WI (**Figure 2B**). In contrast, fat-suppressed T2WI demonstrated a high signal intensity in the abdominal and subcutaneous adiposity region (**Figure 2C**). It seems that the fat was suppressed on T1WI (**Figure 2B**), whereas it was not suppressed on fat-suppressed T2WI (**Figure 2C**). T1WI revealed that the thoracolumbar spinal marrow exhibited slightly high signal intensity, with multiple patches of lower signal intensity dispersed throughout (**Figure 2B**). Conversely, fat-suppressed T2WI demonstrated that the thoracolumbar spinal marrow was inhomogeneous high signal intensity (**Figure 2C**). However, the metastatic tumor located in the spinous process of T12 was not clearly visualized, as indicated by the red arrow in the MRI image (**Figures 2B, C**). Radionuclide bone scintigraphy revealed abnormal areas of high radiotracer uptake in several bone locations, specifically on the left side of the second anterior rib, the right fourth to sixth ribs, and the T12 vertebra along with its appendages. In contrast, the remaining skeleton showed no significant areas of abnormal increased uptake (**Figure 2D**).

The abdominal CT scan indicated that the L3 skeletal muscle index was 50.70 cm²/m² 24 months prior (May 2022) (**Figure 2E**), 39.41 cm²/m² 18 months prior (December 2023) (**Figure 2F**), and 37.89 cm²/m² one month prior (April 2024) (**Figure 2G**). Concurrently, the subcutaneous fat index was recorded as 30.43 cm²/m² (**Figure 2E**), 5.47 cm²/m² (**Figure 2F**), and 0 cm²/m² (**Figure 2G**), respectively, while the visceral fat index was 27.20 cm²/m² (**Figure 2E**), 3.23 cm²/m² (**Figure 2F**), and 0 cm²/m² (**Figure 2G**), respectively. The abdominal cavity and subcutaneous fat regions showed a decrease in volume, while their densities increased, ultimately resulting in a fluid-like density as indicated by the red arrow in the CT image (**Figures 2E–G**). The lower back muscles exhibited gradual atrophy accompanied by fatty infiltration (**Figures 2E–G**).

Diagnosis of SABM was confirmed by two independent radiologists using established MRI criteria (“flip-flop” effect), consistent with prior literature (4). Biopsy was deemed unnecessary given pathognomonic imaging features.

## 3 Discussion

In our SABM case study, we observed a gradual reduction and eventual depletion of both subcutaneous and visceral fat tissue, alongside fluid-like density in these areas in the CT images. The fluid-like density of the fat tissue region may represent the consumption of fat. SABM results from prolonged negative energy balance, causing lipid mobilization and gelatinous bone marrow transformation (4, 11). Initial malnutrition triggers subcutaneous and visceral fat mobilized to meet energy demands (12, 13). This establishes a vicious cycle in which fat-driven inflammation and tumor consumption of metabolites promote muscle degradation, whose resulting energy deficit in turn accelerates further fat catabolism, ultimately fueling tumor growth (14–16). This pattern of progressive fat depletion and muscle wasting reflects a state of severe catabolism. In extreme cases where both subcutaneous and visceral fat tissue have decreased significantly and transformed into a fluid-like density, the occurrence of SABM should be highly suspected, warranting an MRI scan for diagnosis in these patients. To our knowledge, this detailed longitudinal quantification of progressive total body fat depletion to complete absence on CT has not been previously emphasized in SABM literature (4, 13, 17).

As the condition progresses, the depletion of fat moves from subcutaneous and visceral stores to bone marrow fat. The involvement of marrow fat, often occurring later, signifies a more advanced stage of the disease. SABM primarily affects the extremities’ bones, differing from other bone marrow disorders (4, 17). The involvement of the bone’s middle shaft indicates a more advanced stage. MRI results show that on T1WI, the affected bone marrow’s signal intensity is lower than that of skeletal muscle, with no high signals from adjacent subcutaneous and visceral fat. In contrast, fat suppression-T2WI reveals heterogeneous high signal intensity in the affected marrow, while adjacent surrounding fat showed similar high signal changes. This phenomenon, referred to as the “flip-flop” effect in other study, that T1WI looks like fat being suppressed, while fat-suppressed T2WI looks like fat not being suppressed (6, 7, 18, 19). SABM starts with small focal lesions that evolve into diffuse changes, typically lacking enhancement on contrast-enhanced MRI due to the presence of hyaluronic acid-rich gelatinous matrix, unlike tumors or infections (4, 6, 20). In our two cases, DWI revealed no restricted diffusion for Case 1, and bone scans for Case 2 exhibited no abnormal high uptake in the majority of the bone marrow throughout the body, indicating the presence of inactive bone marrow disease. In the appropriate clinical context, the combination of systemic fat depletion on CT and the typical ‘flip-flop’ sign on MRI is highly suggestive of SABM and can often obviate the need for invasive biopsy (4).

In case 2, the spinous process of T12 metastases exhibits poor contrast with surrounding structures on MRI; however, CT revealed significant lytic bone destruction. It has been suggested that SABM tends to lead to underdiagnosis of fractures (4). In these circumstances, detecting bone metastases is especially challenging. The challenge of detecting bone metastases within the context of SABM has, to our knowledge, received limited attention in the existing literature (4, 6, 7, 17, 21). Therefore, in the context of SABM, CT and MRI play complementary roles.

SABM serves as a crucial marker of severe systemic disease, prompting timely evaluation of underlying conditions such as malnutrition associated with malignancy. Longitudinal CT-derived body composition parameters offer objective indicators of a patient’s deteriorating nutritional status (9). Early identification of these changes facilitates the implementation of appropriate clinical management strategies, including nutritional interventions and fracture prevention (5). Additionally, case reports indicate that the administration of granulocyte colony-stimulating factor (G-CSF) promotes the proliferation of granulocyte precursors, thereby contributing to the reversal of SABM (22, 23).

In summary, this case report presents two cases of SABM, both of which were postoperative gastric cancer patients suffering from severe malnutrition. The complete absence of both subcutaneous and visceral fat tissue, as evaluated by longitudinal CT-derived body composition parameters, should raise strong suspicion of SABM.

## Data Availability

Publicly available datasets were analyzed in this study. This data can be found here: The data supporting the findings presented in this case report are available within the article, with patient identifiers removed to ensure confidentiality.
